# Leucine–Histidine Dipeptide Attenuates Microglial Activation and Emotional Disturbances Induced by Brain Inflammation and Repeated Social Defeat Stress

**DOI:** 10.3390/nu11092161

**Published:** 2019-09-09

**Authors:** Yasuhisa Ano, Masahiro Kita, Shiho Kitaoka, Tomoyuki Furuyashiki

**Affiliations:** 1 Research Laboratories for Health Science & Food Technologies, Kirin Holdings Company Ltd, Kanazawa-ku, Yokohama-shi, Kanagawa 236-0004, Japan; 2 Division of Pharmacology, Kobe University Graduate School of Medicine, Kobe 650-0017, Japan (S.K.) (T.F.); 3 Japan Agency for Medical Research and Development (AMED)-CREST, Chiyoda-ku, Tokyo 100-0004, Japan

**Keywords:** antidepressant, cytokine, dipeptide, inflammation, microglia

## Abstract

The number of patients with mental illnesses is rapidly increasing, and daily lifestyle is closely associated with the development of symptoms. It is suggested that inflammatory molecules derived from microglia play crucial roles for the pathophysiology of depression. In the present study, we discovered that leucine–histidine (LH) dipeptide suppressed activation of primary microglia. The effects of LH dipeptide orally administered were measured using tail suspension test (TST) in mice injected with lipopolysaccharide and social interaction test in mice received social defeat stress. LH dipeptide reduced pro-inflammatory cytokines upon stimulation in microglia. Orally administered LH dipeptide was delivered to the brain and suppressed the production of pro-inflammatory cytokines in the brain and concomitant depression-like behavior in the TST. Moreover, oral administration of LH dipeptide suppressed the induction of depression- and anxiety-like behaviors induced by repeated social defeat stress. These results indicate that LH dipeptide suppressed the activation of microglia and ameliorated depression-associated emotional disturbances. Further, we found that LH dipeptide was abundant in various fermented products. Together with previous epidemiological reports that daily intake of these fermented foods is negatively associated with the incidence of psychiatric diseases, our findings suggest that food rich in LH dipeptide may improve mental health.

## 1. Introduction

Depression, which is one of the most common serious psychiatric diseases worldwide [1], is characterized by the dysregulation of emotion and mood that are associated with abnormalities such as cognition, sleep, appetite, and metabolism. Moreover, depressive disorder is a leading cause of disability [2,3]. Unfortunately, numerous patients do not respond to available pharmacological or psychological treatments, and >50% of patients do not respond to first-line pharmacological intervention [4,5]. Thus, the development of novel antidepressants, including prevention using nutritional approaches in daily life, is receiving increasing attention [6,7].

Bioactive peptides are proteolytically released from naturally occurring food proteins. Evidence indicates that some dipeptides improve memory, ameliorate depression, and relieve anxiety. For example, a pyroglutamyl–leucine dipeptide exerts antidepressant effects [8], a tyrosine–leucine dipeptide acts as an anxiolytic with activity comparable with that of diazepam [9], and a tryptophan–tyrosine dipeptide ameliorates deficiencies in memory [10]. Further, an epidemiological study found that consumption of low-fat dairy products, including fermented products, is inversely associated with the symptoms of depression [11,12]. Together, these findings strongly suggest that certain dietary components produced during fermentation, such as peptides and amino acids, contribute to the prevention of depression.

Clinical studies have suggested a role of inflammation in depression. It has been reported that the levels of many inflammation-related molecules are upregulated in the blood, and less frequently in the cerebrospinal fluid, of depressive patients [13,14]. In the central nervous system, microglia play an important role to regulate inflammatory responses. Generally, microglia remove the waste products and old synapses accompanied with aging and promote new synapse formation, but massively activated microglia generated inflammatory cytokines and reactive species oxygen, which are toxic for the neuron [15,16,17]. Recent studies showed that depression is associated with the activation of microglia and stress [18,19]. So, to regulate the activation of microglia is important to keep brain function. To prevent brain disease, there has been increasing attention in the regulation of microglia.

Meta-analyses of randomized controlled trials have indicated that non-steroidal anti-inflammatory drugs (NSAIDs) and anti-tumor necrosis factor (TNF) treatments may improve the symptoms of depression [20,21]. Brain PET imaging shows elevated signals of translocator protein, an inflammation marker, in the anterior cingulate cortex of patients with depression and suicidal thoughts. This indicates the occurrence of inflammation in the brain of depressive patients. Rodent studies have shown that chronic environmental stress, such as chronic mild stress and repeated social defeat stress (R-SDS), causes microglial activation and infiltration of monocytes into the brain, which is crucial for chronic stress-induced emotional disturbances [18,22,23,24,25,26,27]. It has also been shown that the innate immune receptors toll-like receptor (TLR) two and four mediate R-SDS-induced microglial activation in the medial prefrontal cortex, thereby generating depression-like behavior through inflammatory cytokines, such as TNF-α [18]. These basic and clinical studies suggest that brain inflammation is a promising target for the development of preventive and therapeutic agents for depression. Unfortunately, the nutritional components that suppress activation of microglia and contribute to the prevention of depression have not been elucidated well.

Here we evaluated the effects of 336 dipeptides on the activation of microglia and found that a leucine–histidine (LH) dipeptide was a potent anti-inflammatory agent. Therefore, we conducted a behavioral study to evaluate the antidepressant activity of the LH dipeptide.

## 2. Materials and Methods 

### 2.1. Peptides

We purchased 336 dipeptides (Dipeptide Library, AS-65123-336) from ANASpec (CA, USA). These dipeptides (50 μM) were used for in vitro experiments. Leu–His (LH) dipeptides for in vitro and in vivo assays were purchased from ANASpec (purity > 95%) and Kokusan Chemical (purity > 90%, Tokyo, Japan), respectively.

### 2.2. Animals

Pregnant C57BL/6J mice and six-week-old male ICR mice (Charles River Japan, Tokyo, Japan) were maintained at the Kirin Company Ltd. Nine-week-old male C57BL/6N mice and ICR mice (retired breeders) (Japan SLC, Shizuoka, Japan), which were used in the experiments to assess SDS, were maintained at Kobe University or Kirin Company Ltd. Seven-week-old Sprague–Dawley (SD) rats (Charles River Japan) were maintained at Sekisui Medical Ltd. All experiments using mice and rats were approved by the Animal Experiment Committee of Kobe University, Kirin Company Ltd., and Sekisui Medical Ltd., and conducted in strict accordance with each of their guidelines since 2016 to 2017 (Approval ID; AN10145-Z00 AN10200-Z00, AN10253-Z00). Mice were euthanized by placing them in a chamber filled with isoflurane gas (Wako, Tokyo, Japan), and rats anesthetized with isoflurane were euthanized by exsanguination from the abdominal aorta. Mice were fed a standard rodent diet (CE-2, CLEA Japan, Tokyo, Japan) and maintained at room temperature (23 °C ± 1 °C) under constant 12 h light/dark cycles (light period from 8:00 am to 8:00 pm). All efforts were made to minimize suffering.

### 2.3. Culture of Primary Mouse Microglia

Primary microglia were isolated from the brains of newborn C57BL/6J mice (<seven days of age) using magnetic cell sorting with anti-CD11b antibodies (Miltenyi Biotec, Bergisch Gladbach, Germany) as previously described [28]. CD11b-positive cells (> 90% purely evaluated using flow cytometry) were plated in poly-D-lysine (PDL)-coated 96-well plates (BD Biosciences, Billerica, MA, USA) and cultured in DMEM/F-12 medium (Gibco, Thermo Fisher Scientific, Waltham, MA, USA) supplemented with 10% fetal calf serum (Gibco) and 100 U/mL penicillin/streptomycin (Sigma-Aldrich). Microglia isolated from newborn C57BL/6J mice were plated at 3 × 10^4^ cells/well in a PDL-coated plate.

To measure the production of inflammatory cytokines released into the culture medium, microglia were treated with each peptide for 12 h and then with lipopolysaccharide (LPS) (5 ng/mL; Sigma-Aldrich, Saint Louis, MO, USA) and interferon-γ (IFN-γ) (0.5 ng/mL, R&D Systems, Minneapolis, MN, USA) for 12 h. After treatment, supernatants were tested using an enzyme-linked immunosorbent assay (ELISA) (eBioscience, San Diego, CA, USA) or a multiplex cytokine assay (Bio-Rad, Hercules, CA, USA).

To measure the expression of cell surface markers, microglia were treated with a peptide and then with 5 ng/mL LPS and 0.5 ng/mL IFN-γ. The cells were incubated with the antibodies as follows: Anti-CD86-PE (clone; B7-2, eBioscience), anti-CD206-FITC (clone; C068C2, BioLegend, San Diego, CA, USA), and anti-CD11b-APC-Cy7 (clone; M1/70, BD Biosciences). Flow cytometry was performed using a FACSCanto II flow cytometer (BD Biosciences). Each sample was assayed in triplicate.

### 2.4. LPS Induction of Neuronal Inflammation

Male ICR mice aged six weeks were orally administered with distilled water with or without the LH dipeptide once daily for seven days via a gastric feeding tube, deeply anesthetized with sodium pentobarbital (Kyoritsu Seiyaku, Tokyo, Japan) 30 min after the final administration, and then intracerebroventricularly injected with 10 μg of LPS from *Salmonella typhosa* (L7895, Sigma-Aldrich), as previously reported [29]. Briefly, LPS or phosphate-buffered saline (PBS) (for sham-operated controls) was injected into the right and left cerebral ventricle (5 μL per site). A microsyringe with a 27-gage stainless steel needle, 2 mm long, was used for microinjection. The needle was inserted 1 mm to the right and left of the midline point equidistant from each eye, at an equal distance between the eyes and the ears and perpendicular to the plane of the skull (anteroposterior, −0.22 mm from the bregma). LPS was delivered gradually within 30 s. The needle was removed 30 s later. Distilled water with or without the 50 mg/kg LH dipeptide was orally administered again after 24 h, and 1 h later, the hippocampus and frontal cortex in hemisphere were removed and homogenized in Tris-buffered saline buffer (Wako) containing a protease inhibitor cocktail (BioVision, Milpitas, CA, USA) with a multi-beads shocker (Yasui Kikai, Osaka, Japan). The supernatant was collected after centrifugation at 50,000 × g for 20 min. The protein concentration of each supernatant was measured using a BCA protein assay kit (ThermoScientific, Yokohama, Japan). We used an ELISA (eBioscience) to measure the production of inflammatory cytokines and chemokines in the brain. The amounts of cytokines and chemokines in the supernatants were quantified using an ELISA (eBioscience).

In the other experiment designed to evaluate behavior, samples were orally administered to mice daily for seven days and then 0.5 mg/kg of LPS was intracerebroventricularly injected. The mice were orally administered each sample 24 h after LPS treatment; after 1 h, mice were first subjected to the tail suspension test (TST) to evaluate immobility time during a 6 min interval and then, 30 min later, administered the open field test (OFT) to evaluate locomotor activity.

### 2.5. TST

The TST serves as a model of depression-like behavior and assesses behavioral despair, a component of depression [30]. Each mouse was suspended in a box for 6 min with its tail secured using adhesive tape. A video was recorded, which was carefully scored for the immobility time. Mice were considered immobile only when they hung passively and were completely motionless.

### 2.6. OFT

To measure locomotor activity, mice were subjected to the OFT for 5 min. The distance in the open field box (40 cm by 40 cm by 40 cm, gray polyvinyl chloride) was measured using SMART video tracking software (PanLab Harvard Apparatus, Holliston, MA, USA).

### 2.7. Repeated SDS (R-SDS)

Mice were subjected to R-SDS as previously described [31]. Before R-SDS, ICR male mice were screened for their aggressiveness against a different C57BL/6N mouse for three minutes daily for three days. We evaluated the aggression of ICR mice by measuring latency and the number of attacks during the observation period. We used only mice that exhibited stable aggression. Before administering R-SDS, male C57BL/6N mice were individually housed with free access to food and water for one week. Mice were intragastrically administered 50 mg/kg of the LH dipeptide daily for three days or fed a standard rodent diet AIN-93M containing 0.15% (w/w) LH dipeptide for 10 days before R-SDS. Mice were orally administered or fed dietary LH dipeptide during R-SDS. These mice were then transferred to the home cage of a male ICR mouse for 10 min daily for four or 10 days for those orally administered or fed the LH peptide, respectively. The pairs of defeated and aggressor mice were randomized daily to minimize the variability in the aggressiveness of the aggressor mice. After the 10 min defeat episode, the mice were returned to their home cages and isolated until being subjected to R-SDS on the next day. Control mice were instead transferred to a new cage and were allowed to freely explore for 10 min. We included all data in the analyses.

### 2.8. Social Interaction Test (SIT)

After undergoing R-SDS, defeated and control mice were tested using the social interaction test (SIT) as previously described [31,32]. Briefly, the mice were placed in an open field chamber where a new male ICR mouse was enclosed in a metal meshwork at one end. The mice were allowed to freely explore the chamber for 150 s during recording. The mice were habituated to the same chamber in the absence of an ICR mouse for 150 s before the SIT. The chamber was divided into an interaction zone (closest to the ICR mouse), a middle zone, and an avoidance zone (farthest from the ICR mouse). The time each mouse spent in each zone was subsequently analyzed using SMART video tracking software (PanLab, Harvard Apparatus).

### 2.9. Elevated Plus Maze (EPM) Test

After the final episode of R-SDS, defeated mice were subjected to the elevated plus maze (EPM) test. The EPM comprised two open (30 cm by 7 cm) and two closed (30 cm by 7 cm) arms with walls (20 cm by 30 cm) surrounding the closed arms. The area of the center region was 7 cm by 7 cm. The EPM was placed 50 cm from the ground. The mice were individually placed in the center region and allowed to freely explore for five minutes. The time that each mouse spent and the number of entries to open arms were subsequently analyzed using SMART video tracking software (PanLab).

### 2.10. Pharmacokinetics of the ^14^C-LH Peptide

Carboxyl-^14^C Leu–His (^14^C-LH) was prepared by Sekisui Medical Ltd. Its radiochemical purity was 97.1%, measured using HPLC, and its specific radioactivity was 7.90 MBq/mg. The test solution of 0.2 mg/mL of ^14^C-LH dissolved in distilled water was prepared immediately before administration. SD rats (*n* = 3) were starved for 16 h and orally administered the radioactive dipeptide (2 mg/kg, 10 mL/kg body weight). Plasma samples were collected 2, 5, 10, and 30 min and 1, 2, 4, 6, 8, 10, 24, 48, and 72 h after administration. Radioactivity was quantified using a liquid scintillation counter (HIONIC-FLUOR, PerkinElmer, Waltham, MA, USA). We prepared organ homogenates two hours after administration, and radioactivity was quantified according to a published method [10].

### 2.11. Statistical Analysis

The data represent the mean, and the error bars indicate the SEM. Data were analyzed using one-way ANOVA followed by the Tukey–Kramer test, Dunnett’s test, and Student *t* test, as described in the figure legends. All statistical analyses were performed using Ekuseru-Toukei 2012 software (Social Survey Research Information, Tokyo, Japan) or GraphPad Prism 7 (GraphPad Software, Inc, San Diego, CA, USA). *p* < 0.05 was considered statistically significant.

## 3. Results

### 3.1. Effects of the LH Dipeptide on Primary Microglia

**Figure 1:**
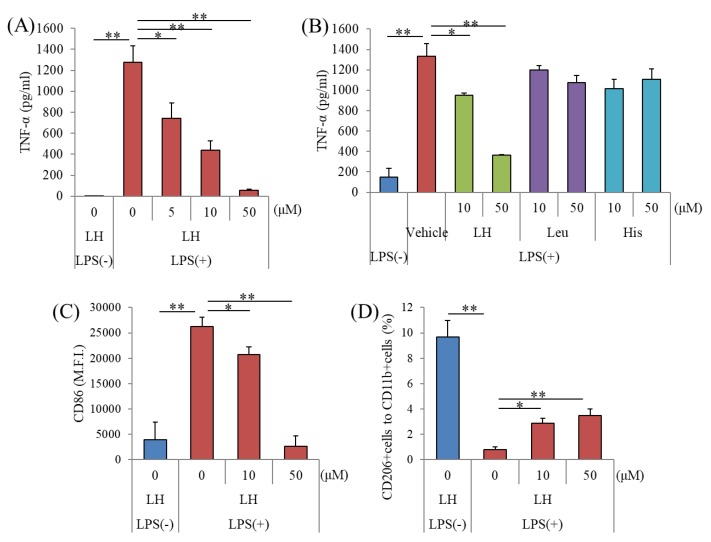
Effects of leucine–histidine (LH) dipeptide on the activation of primary microglia. The effects of in vitro LH dipeptide treatment on inhibiting microglial activation. (**A**), Amount of TNF-α in the supernatant of microglia treated with 0, 5, 10, or 50 μM LH dipeptide followed by treatment with 5 ng/mL lipopolysaccharide (LPS) and 0.5 ng/mL interferon-γ (IFN-γ). (**B**), Amount of TNF-α in the supernatant of microglia treated with 0, 10, or 50 μM LH dipeptide, leucine (Leu), or histidine (His) and followed by treatment with 5 ng/mL LPS and 0.5 ng/mL IFN-γ. (**C**,**D**), The expression of CD86 and percentages of CD206-positive/CD11b-positive microglia treated with 0, 10, or 50 μM LH dipeptide followed by treatment with 5 ng/mL LPS and 0.5 ng/mL IFN-γ, respectively. M.F.I. means mean fluorescent intensity. Columns and bars represent the mean and SE values of three independent wells per sample, respectively. The p values shown were calculated using the Student t test (LPS [−] versus [+], 0 μM LH dipeptide) and one-way ANOVA followed by Dunnett’s test. * *p* < 0.05 and ** *p* < 0.01.

To evaluate the anti-inflammatory effects of dipeptides, microglia were treated with 336 dipeptides (50 μM each) and then treated with LPS. The levels of TNF-α in the supernatants of microglial cultures treated with the LH dipeptide were significantly reduced compared with microglia treated with the other dipeptides (Supplement Figure S1). The LH dipeptide (5 μM) decreased the levels of TNF-α, although leucine and histidine did not (Figure 1A,B). Flow cytometric analysis revealed that the level of the costimulatory factor CD86 was significantly increased by LPS treatment and reduced by the treatment with LH dipeptide (Figure 1C). The percentages of CD206-positive microglia reminiscent of M2-like microglia were significantly increased by treatment with the LH dipeptide (Figure 1D). Moreover, multiplex analysis showed that the levels of proinflammatory cytokines and chemokines (IL-1α, IL-1β, MCP-1, MIP-1α, MIP-1β, and TNF-α) in the supernatants of cell cultures sequentially treated with the LH dipeptide (10 μM) and LPS were lower compared with those in microglia treated with only LPS (Table 1).

### 3.2. Penetration of the LH Dipeptide through the Blood–Brain Barrier

To analyze the pharmacokinetics of the LH dipeptide, ^14^C-LH dipeptide was administered to rats, and radioactivity in the blood was subsequently measured. To measure the time course of absorption of LH dipeptide into body at 15 time points, we used rats. The ^14^C-LH dipeptide was detected in blood as early as 2 min after oral administration, and its concentration was estimated to reach a maximum at 1.67 h (Table 2). The tissue distribution of radioactivity 2 h after oral administration shows that the ratios of tissue-to-plasma concentrations were approximately 0.2 in all the brain regions tested (Table 3). Thus, there were no differences in the distributions of radioactivity among different regions of the brain. These results suggest that the LH dipeptide or its metabolites penetrated the blood–brain barrier. However, the radioactivity from the ^14^C-LH dipeptide was measured but LC/MS/MS measurements were not performed, which limits the strength of the conclusion that the radioactivity detected in each organ corresponded to the naïve ^14^C-LH dipeptide or its metabolites.

### 3.3. Effects of the LH Dipeptide on Inflammation in the Brain

**Figure 2:**
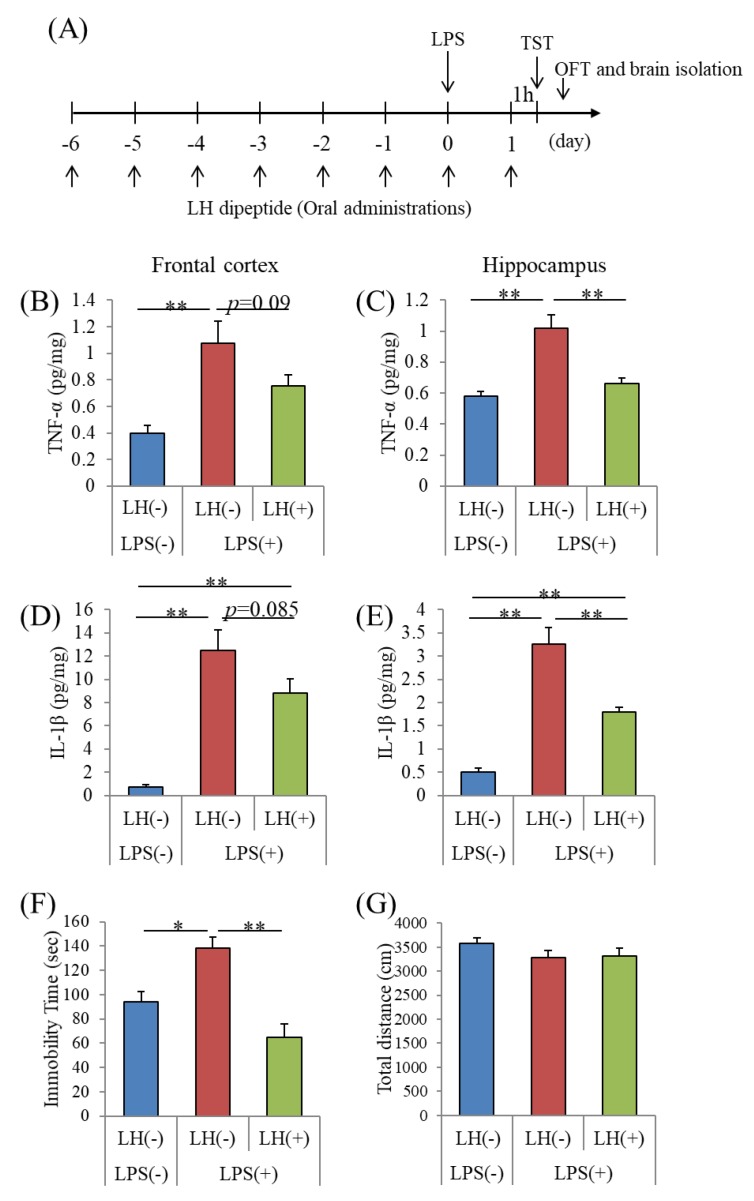
Effects of LH dipeptide on neuronal inflammation and depression-like behavior induced by LPS. (**A**), ICR mice were orally administered 0 or 50 mg/kg of LH dipeptide for seven days and then intracerebroventricularly injected with PBS or 0.5 mg/kg LPS 1 h after the last administration. The mice were subjected to the tail suspension test (TST) and open field test 24 h after the administration of LPS. B-E, Amounts of TNF-α in frontal cortex (**B**) and hippocampus (**D**) and IL-1β in the frontal cortex (**C**) and hippocampus (**E**) 24 h after LPS injection, respectively. (**F**), Immobility timed in the TST. (**G**), Total distances (cm) in the open field test. H, Immobility times in the TST of sham-operated mice. Data represent the mean ± SE of 10 mice per group. The p values were calculated using one-way ANOVA followed by the Tukey–Kramer test. *p < 0.05 and **p < 0.01.

Next, we examined whether repeated oral administration of the LH dipeptide suppressed neural inflammation induced by intracerebroventricular treatment with LPS (Figure 2A). Intracerebroventricular treatment with LPS significantly increased the amounts of TNF-α and IL-1β in the frontal cortex (Figure 2B,D, respectively) and hippocampus (Figure 2C,E, respectively). Repeated oral administration of the LH dipeptide (50 mg/kg) reduced the amounts of TNF-α and IL-1β in hippocampus (Figure 2C,E, respectively). The treatment with the LH dipeptide also tended to reduce the amounts of these cytokines in the frontal cortex (Figure 2B,D, respectively). These results indicate that the orally administered LH dipeptide suppressed inflammation in the brain. Prior to this experiment, we found that repeated oral administration of LH at 10 and 50 mg/kg suppressed the production of cerebral cytokines generated by intracerebroventricular LPS treatment, in a dose-dependent manner (data not shown).

### 3.4. Effects of the LH Dipeptide on Depression-Associated Emotional Disturbances

To evaluate the effects of the LH dipeptide on depression-associated emotional disturbances, we first used the time for immobility in the TST. After repeated oral administration with the LH dipeptide, mice were treated intracerebroventricularly with LPS and then subjected to the TST (Figure 2A). Intracerebroventricular treatment with LPS increased immobility time, which was inhibited by prior treatment with the LH dipeptide (Figure 2F). Neither LPS nor the LH dipeptide significantly affected locomotor activity in the OFT (Figure 2G).

**Figure 3:**
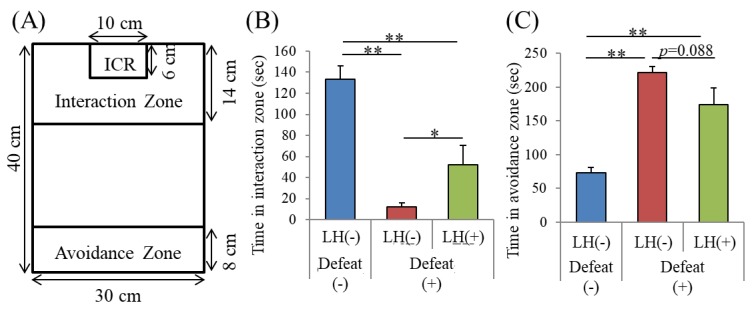
Effects of intragastrically administered LH dipeptide on repeated social defeat stress (R-SDS). C56BL/6N mice were orally administered 50 mg/kg LH dipeptide and subjected to R-SDS daily for 10 consecutive days. (**A**), On days 0, 2, and 4, mice were subjected to social interaction with ICR mice in chamber. (**B**), Times in the interaction zone on days 2 of R-SDS. (**C**), Times in the avoidance zone on days 2 of R-SDS. Data represent the mean ± SE of 8–10 mice per group. The p values were calculated by one-way ANOVA followed by the Tukey–Kramer test. * *p* < 0.05 and ** *p* < 0.01.

**Figure 4:**
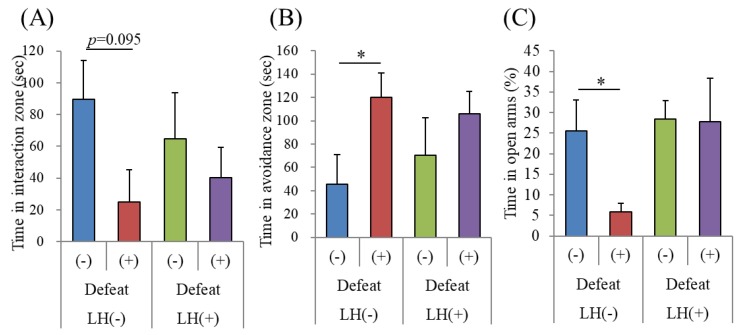
Effects of dietary LH dipeptide on R-SDS. C56BL/6N mice were supplied with a diet containing 0.15% LH dipeptide and subjected to R-SDS daily for 10 consecutive days. (**A**), On days 0, 2, and 6, mice were subjected to social interaction with ICR mice in the chamber. (**B**), Times in the interaction zone on days 6. (**C**), Times in the avoidance zone on days 6. After 11 days, mice were subjected to the elevated plus maze for 5 min, and times spent in the open arms were measured. C, The percentage of time in open arms. Data represent the mean ± SE of four to eight mice per group. The p values were calculated using the Student t test to evaluate the significance of the difference between defeat (−) and (+) with LH (−) or without LH (+). * *p* < 0.05.

We then examined the effects of the LH dipeptide on the emotional disturbances induced by R-SDS, a non-inflammatory mouse model of depression. The mice were orally treated with the LH dipeptide using gastric needles once daily for consecutive days, during which they were subjected to R-SDS. Unlike what is usually observed, this administration method induced social avoidance as soon as after the second stress exposure. Nonetheless, the orally administered LH dipeptide significantly reduced the induction of social avoidance (Figure 3B,C). Because intragastric administration performed before and during the stress seems to enhance stress susceptibility, a different administration method was used, in which the mice were fed a regular diet, with or without the LH dipeptide, from 10 days prior to the beginning of SDS. With the control diet, R-SDS induced significant social avoidance. On the other hand, the diet containing the LH dipeptide inhibited the induction of social avoidance (Figure 4A,B). Notably, the LH dipeptide-containing diet abolished the induction of elevated anxiety by R-SDS, as measured by the time spent on the open arms of the EPM (Figure 4C). It should be noted that the LH dipeptide-containing diet did not affect the time spent in the open arms without exposure to SDS, suggesting that neither the normal level of anxiety nor locomotor activity was impaired (data not shown). Collectively, these results show that the LH peptide ameliorated the emotional disturbances induced by both inflammatory stimuli and R-SDS.

## 4. Discussion

In the present study, we discovered that the LH dipeptide suppressed the inflammatory response in primary microglia. When orally administered, the LH dipeptide reached the systemic circulation and passed through the blood–brain barrier to suppress inflammation in the brain. Such treatment ameliorated LPS-induced depression-like behavior and repeated social defeat stress-induced emotional disturbances. Therefore, the LH dipeptide and food materials enriched in this dipeptide may be useful to promote mental health through suppressing inflammatory responses in the brain.

In vitro assays revealed that the LH dipeptide, but not leucine or histidine, reduced the levels of proinflammatory cytokines but not those of an anti-inflammatory cytokine. We assume that the anti-inflammatory effects of the LH dipeptide depend on the recognition of some receptors. For example, it has been reported that some dipeptides and tripeptides activate the bitter taste receptor (T2R) [33] whereas T2R has been reported to express on myeloid cells [34], so these receptors might be involved in the anti-inflammatory effects. Further studies are required to investigate the underlying mechanisms of the effects of LH dipeptide on suppressing microglial activation. Further, treatment with the LH dipeptide increased the number of CD206-positive microglia following treatment with LPS. Proinflammatory cytokines such as TNF-α are produced by M1 microglia, and CD206 is a marker of M2 anti-inflammatory microglia [35]. M1 microglia are involved in the development of neurological disorders, including depression [36]. In contrast, M2 microglia play important neuroprotection and wound healing roles in neuronal tissues [37]. Our results therefore indicate that the LH dipeptide suppressed M1 microglial response. Moreover, the LH dipeptide suppressed the production of proinflammatory cytokines both by human and murine microglia. Recently, it has been suggested that microglial phagocytosis is involved not only in neuronal remodeling during brain development but also in the neuronal dysfunction involved in the pathophysiology of neurological disorders. While the role of microglial phagocytosis in mental illnesses remains elusive, we previously found that R-SDS increased the signals of CD68, a marker for phagocytic activity, in the prefrontal microglia in a manner correlated to the level of social avoidance. Therefore, whether the LH dipeptide also affects microglial phagocytosis upon stimulation warrants future investigation.

In the present study, oral administration of the LH dipeptide suppressed LPS-induced increase in TNF-α and IL-1β in the hippocampus and, at a lower level, in the frontal cortex. Microglia are one of the major cellular sources of these cytokines in the brain. Based on our finding that the LH dipeptide and its metabolites enter the brain through the blood–brain barrier, the LH dipeptide could directly suppress LPS-induced microglial activation, thereby suppressing the microglial production of these cytokines. Consistent with its effect on neural inflammation, the orally administered LH dipeptide suppressed the LPS-induced immobility time increase in the TST. Notably, the LH dipeptide did not affect the basal level of immobility without LPS treatment, suggesting that the LH dipeptide could spare emotional behaviors in the physiological condition and specifically suppress inflammation-associated pathological changes of emotional behaviors, thus indicating minimal side effects on brain functions. Intracerebroventricular administration of LPS activates microglia and induces dendritic changes in the hippocampus and prefrontal cortex, resulting in depression-like behavior [38,39]. In contrast, inflammation suppression can ameliorate these impairments [38,39]. Because both the hippocampus and prefrontal cortex are involved in depression [40], the inhibitory effects of the LH dipeptide on inflammation in these regions may attenuate inflammation-related symptoms of depression.

To evaluate the effects of the LH dipeptide on depression-like symptoms induced by social stress, we conducted the R-SDS test, because in our previously published data, the depression-like behavior in the R-SDS test was associated with activation of microglia in the brain [18]. Consistent with the effects of the LH dipeptide in suppressing microglial activation, the orally administered LH dipeptide suppresses the induction of social avoidance and elevated anxiety by R-SDS. These results suggest that dietary consumption of the LH dipeptide ameliorated depression and anxiety induced by social stress. We previously reported that R-SDS activates prefrontal microglia through TLR2/4, which in turn induces social avoidance through proinflammatory cytokines, such as TNF-α. It has also been shown that chronic or repeated environmental stress induces microglial activation in the hippocampus, which suggests that production of IL-1β and impaired adult neurogenesis are potential behavior change mechanisms. Thus, the LH dipeptide may attenuate inflammatory responses simultaneously in several brain areas. This study shows that the LH dipeptide suppresses microglial activation. 

The current study contains some limitations. First, we did not evaluate whether detection of radioactivity in the brain is ^14^C-LH dipeptide, its metabolites, or leucine using LC/MS/MS. Histidine and leucine did not suppressed the TNF-α production by primary microglia, so it is suggested that LH dipeptide or its metabolites contribute to suppress the activation of microglia. This point is important to elucidate the underling mechanisms. This limitation needs to be solved in further studies. Second, LH dipeptide could directly affect the functional and structural properties of neurons. To elucidate the LH dipeptide mechanism of action, it is crucial to identify a putative receptor(s) for the LH dipeptide. Third, we need to evaluate whether the LH dipeptide suppresses microglial activation induced by R-SDS in future investigation.

## 5. Conclusions

In conclusion, the current study suggested that intakes with LH dipeptide might contribute to the prevention of depression or inflammatory-related mood disorders. Further studies are required to evaluate the effects of supplementation of LH dipeptide or food materials rich in LH dipeptide on human depression or psychological stress.

## Figures and Tables

**Table 1:** Cytokine and chemokine levels in the supernatants of microglial cultures.

	LPS(−)	LPS(+)	LPS(+)	LPS(+)
LH	0 μM	0 μM	10 μM	30 μM
	Mean ± SE	Mean ± SE	Mean ± SE	Mean ± SE
IL-1α	10.85±0.41	33.92 ± 4.52 ^##^	27.80 ± 0.35*	21.03 ± 2.76 *
IL-1β	N.D.	461.90 ± 22.31	370.28 ± 7.15*	232.69 ± 65.04 *
IL-2	0.49±0.16	5.11 ± 0.85 ^##^	3.83 ± 0.68	1.96 ± 1.20 *
IL-4	11.03±1.87	21.10 ± 7.26	16.87 ± 2.39	10.92 ± 2.53
IL-5	N.D.	70.64 ± 6.00	59.81 ± 3.63	34.33 ± 2.96 *
IL-6	1.49 ± 0.17	1115.34 ± 291.93 ^##^	868.39 ± 63.81	40.64 ± 4.66 **
IL-9	N.D.	0.97 ± 0.51	0.95 ± 0.48	N.D.
IL-10	4.42 ± 0.88	46.67 ± 4.41 ^##^	32.60 ± 2.41	9.81 ± 6.38 *
IL-12p40	3.67 ± 0.05	2814.93 ± 1672.56 ^##^	1285.46 ± 64.50	26.13 ± 6.46 **
IL-12p70	31.41 ± 7.59	274.67 ± 70.58 ^##^	255.40 ± 20.96	114.16 ± 48.49
IFN-γ	N.D.	203.20 ± 43.29	200.09 ± 13.56	205.37 ± 11.01
KC	249.36 ± 21.45	608.00 ± 325.53	617.92 ± 54.11	164.15 ± 15.42 *
G-CSF	14.82 ± 0.89	2553.57 ± 858.49 ^#^	1526.14 ± 213.37	35.49 ± 7.38 **
GM-CSF	3.24 ± 0.70	12.48 ± 3.77 ^#^	15.78 ± 1.46	6.10 ± 1.73
Eotaxin	N.D.	115.55 ± 114.07	5.41 ± 2.50	N.D.
MCP-1	341.31 ± 2.31	6309.88 ± 874.11 ^#^	4575.50 ± 224.07 *	245.64 ± 48.90 **
MIP-1α	1456.73 ± 57.27	7558.73 ± 441.63 ^##^	4338.73 ± 1422.37 **	1167.67 ± 273.58 **
MIP-1β	1750.02 ± 82.41	34440.79 ± 4285.71 ^##^	22361.34 ± 2599.65 *	1674.58 ± 42.01 **
RANTES	211.50 ± 3.28	13515.62 ± 6828.57 ^##^	18915.28 ± 1333.45	3393.04 ± 1229.12
TNF-α	N.D.	4239.37 ± 688.90	758.51 ± 115.39 **	369.73 ± 84.86 **

Levels of cytokines and chemokines in the supernatants of primary microglial cultures treated with 0, 10, or 30 μM LH dipeptide followed by treatment with 5 ng/mL LPS and 0.5 ng/mL IFN-γ. The values represent the mean and SE of triplicate samples. *P* values were calculated using the Student *t* test (LPS [−] versus [+] at 0 μM LH dipeptide) and one-way ANOVA followed by Dunnett’s test (LPS [+] at 0 μM LH versus LPS [+] at 10 and 30 μM). *^, #^ *p* < 0.05 and **^, ##^ *p* < 0.01.

**Table 2:** Pharmacokinetics of the LH dipeptide. Plasma radioactivity in seven-week-old SD rats after oral administration of 2 mg/kg (carboxyl-^14^C) Leu–His.

Time	Radioactivity (ng/mL)	
	Mean	SE
2 min	229.7	89.0
5 min	568.5	126.0
10 min	1424.0	167.3
30 min	2006.7	179.4
1 h	3839.5	389.2
2 h	3915.3	292.4
4 h	3860.1	313.9
6 h	3360.5	362.1
8 h	3000.5	215.5
10 h	2776.0	164.2
24 h	1704.9	147.8
48 h	1151.0	89.9
72 h	867.6	37.5
120 h	543.2	17.5
168 h	364.0	20.1
C_max_ (ng eq./mL)	3955.6	574.3
T_max_(h)	1.67	0.58
T_1/2_(h)	76.37	1.91

**Table 3:** Tissue distribution of radioactivity 2 h after oral administration of 2 mg/kg (carboxyl-*14*C) Leu–His. The ratio of tissue-to-plasma concentrations after 2 h is shown.

Tissue	Radioactivity Concentration (ng eq. of LH /g or mL)		
	Mean	SE	Ratio
Plasma	3770.7	728.7	1.00
Cerebrospinal fluid	34.4	1.9	0.01
Cerebrum	703.7	64.3	0.19
Cerebral cortex	788.9	32.4	0.21
Hippocampus	722.4	38.7	0.19
Striatum	661.7	21.1	0.18
Thalamus	702.4	63.1	0.19
Hypothalamus	710.0	130.1	0.19
Midbrain	812.7	42.4	0.22
Cerebellum	704.9	293.5	0.19
Spinal cord	553.1	29.8	0.15
Liver	8207.5	561.5	2.18
Kidney	4023.0	168.2	1.07

## Supplementary Materials

The following are available online at https://www.mdpi.com/2072-6643/11/9/2161/s1, Figure S1: Effects of dipeptides on the production of TNF-α by microglia.

## References

[1] World Health Organization (2016). Depression Fact Sheet. http://www.who.int/en/news-room/fact-sheets/detail/depression.

[2] Tanti A., Belzung C. (2010). Open questions in current models of antidepressant action. Br. J. Pharmacol..

[3] Eisch A.J., Petrik D. (2012). Depression and hippocampal neurogenesis: a road to remission?. Science.

[4] Papakostas G.I. (2009). Managing partial response or nonresponse: switching, augmentation, and combination strategies for major depressive disorder. J. Clin. Psychiatry.

[5] Thase M.E., Friedman E.S., Biggs M.M., Wisniewski S.R., Trivedi M.H., Luther J.F., Fava M., Nierenberg A.A., McGrath P.J., Warden D. (2007). Cognitive therapy versus medication in augmentation and switch strategies as second-step treatments: a STAR*D report. Am. J. Psychiatry.

[6] Opie R.S., Itsiopoulos C., Parletta N., Sanchez-Villegas A., Akbaraly T.N., Ruusunen A., Jacka F.N. (2017). Dietary recommendations for the prevention of depression. Nutr. Neurosci..

[7] Roca M., Kohls E., Gili M., Watkins E., Owens M., Hegerl U., van Grootheest G., Bot M., Cabout M., Brouwer I.A. (2016). Prevention of depression through nutritional strategies in high-risk persons: rationale and design of the MooDFOOD prevention trial. BMC Psychiatry.

[8] Yamamoto Y., Mizushige T., Mori Y., Shimmura Y., Fukutomi R., Kanamoto R., Ohinata K. (2015). Antidepressant-like effect of food-derived pyroglutamyl peptides in mice. Neuropeptides.

[9] Mizushige T., Kanegawa N., Yamada A., Ota A., Kanamoto R., Ohinata K. (2013). Aromatic amino acid-leucine dipeptides exhibit anxiolytic-like activity in young mice. Neurosci. Lett..

[10] Ano Y., Ayabe T., Kutsukake T., Ohya R., Takaichi Y., Uchida S., Yamada K., Uchida K., Takashima A., Nakayama H. (2018). Novel lactopeptides in fermented dairy products improve memory function and cognitive decline. Neurobiol. Aging.

[11] Cui Y., Huang C., Momma H., Ren Z., Sugiyama S., Guan L., Niu K., Nagatomi R. (2017). Consumption of low-fat dairy, but not whole-fat dairy, is inversely associated with depressive symptoms in Japanese adults. Soc. Psychiatry Psychiatr. Epidemiol..

[12] Miyake Y., Tanaka K., Okubo H., Sasaki S., Arakawa M. (2015). Intake of dairy products and calcium and prevalence of depressive symptoms during pregnancy in Japan: a cross-sectional study. BJOG Int. J. Obstet. Gynaecol..

[13] Syed S.A., Beurel E., Loewenstein D.A., Lowell J.A., Craighead W.E., Dunlop B.W., Mayberg H.S., Dhabhar F., Dietrich W.D., Keane R.W. (2018). Defective Inflammatory Pathways in Never-Treated Depressed Patients Are Associated with Poor Treatment Response. Neuron.

[14] Lindqvist D., Janelidze S., Hagell P., Erhardt S., Samuelsson M., Minthon L., Hansson O., Bjorkqvist M., Traskman-Bendz L., Brundin L. (2009). Interleukin-6 is elevated in the cerebrospinal fluid of suicide attempters and related to symptom severity. Biol. Psychiatry.

[15] Block M.L., Zecca L., Hong J.S. (2007). Microglia-mediated neurotoxicity: uncovering the molecular mechanisms. Nat. Rev. Neurosci..

[16] Hansen D.V., Hanson J.E., Sheng M. (2018). Microglia in Alzheimer’s disease. J. Cell Biol..

[17] Sarlus H., Heneka M.T. (2017). Microglia in Alzheimer’s disease. J. Clin. Investig..

[18] Nie X., Kitaoka S., Tanaka K., Segi-Nishida E., Imoto Y., Ogawa A., Nakano F., Tomohiro A., Nakayama K., Taniguchi M. (2018). The Innate Immune Receptors TLR2/4 Mediate Repeated Social Defeat Stress-Induced Social Avoidance through Prefrontal Microglial Activation. Neuron.

[19] Yirmiya R., Rimmerman N., Reshef R. (2015). Depression as a microglial disease. Trends Neurosci..

[20] Kappelmann N., Lewis G., Dantzer R., Jones P.B., Khandaker G.M. (2018). Antidepressant activity of anti-cytokine treatment: a systematic review and meta-analysis of clinical trials of chronic inflammatory conditions. Mol. Psychiatry.

[21] Kohler O., Benros M.E., Nordentoft M., Farkouh M.E., Iyengar R.L., Mors O., Krogh J. (2014). Effect of anti-inflammatory treatment on depression, depressive symptoms, and adverse effects: a systematic review and meta-analysis of randomized clinical trials. JAMA Psychiatry.

[22] Wohleb E.S., Hanke M.L., Corona A.W., Powell N.D., Stiner L.M., Bailey M.T., Nelson R.J., Godbout J.P., Sheridan J.F. (2011). beta-Adrenergic receptor antagonism prevents anxiety-like behavior and microglial reactivity induced by repeated social defeat. J. Neurosci. Off. J. Soc. Neurosci..

[23] Tanaka K., Furuyashiki T., Kitaoka S., Senzai Y., Imoto Y., Segi-Nishida E., Deguchi Y., Breyer R.M., Breyer M.D., Narumiya S. (2012). Prostaglandin E2-mediated attenuation of mesocortical dopaminergic pathway is critical for susceptibility to repeated social defeat stress in mice. J. Neurosci. Off. J. Soc. Neurosci..

[24] Iwata M., Ota K.T., Li X.Y., Sakaue F., Li N., Dutheil S., Banasr M., Duric V., Yamanashi T., Kaneko K. (2016). Psychological Stress Activates the Inflammasome via Release of Adenosine Triphosphate and Stimulation of the Purinergic Type 2X7 Receptor. Biol. Psychiatry.

[25] Menard C., Pfau M.L., Hodes G.E., Kana V., Wang V.X., Bouchard S., Takahashi A., Flanigan M.E., Aleyasin H., LeClair K.B. (2017). Social stress induces neurovascular pathology promoting depression. Nat. Neurosci..

[26] McKim D.B., Weber M.D., Niraula A., Sawicki C.M., Liu X., Jarrett B.L., Ramirez-Chan K., Wang Y., Roeth R.M., Sucaldito A.D. (2018). Microglial recruitment of IL-1beta-producing monocytes to brain endothelium causes stress-induced anxiety. Mol. Psychiatry.

[27] Wohleb E.S., Terwilliger R., Duman C.H., Duman R.S. (2018). Stress-Induced Neuronal Colony Stimulating Factor 1 Provokes Microglia-Mediated Neuronal Remodeling and Depressive-like Behavior. Biol. Psychiatry.

[28] Ano Y., Dohata A., Taniguchi Y., Hoshi A., Uchida K., Takashima A., Nakayama H. (2017). Iso-alpha-acids, Bitter Components of Beer, Prevent Inflammation and Cognitive Decline Induced in a Mouse Model of Alzheimer’s Disease. J. Biol. Chem..

[29] Ano Y., Ozawa M., Kutsukake T., Sugiyama S., Uchida K., Yoshida A., Nakayama H. (2015). Preventive effects of a fermented dairy product against Alzheimer’s disease and identification of a novel oleamide with enhanced microglial phagocytosis and anti-inflammatory activity. PLoS ONE.

[30] Can A., Dao D.T., Terrillion C.E., Piantadosi S.C., Bhat S., Gould T.D. (2012). The tail suspension test. J. Vis. Exp. JoVE.

[31] Shinohara R., Taniguchi M., Ehrlich A.T., Yokogawa K., Deguchi Y., Cherasse Y., Lazarus M., Urade Y., Ogawa A., Kitaoka S. (2018). Dopamine D1 receptor subtype mediates acute stress-induced dendritic growth in excitatory neurons of the medial prefrontal cortex and contributes to suppression of stress susceptibility in mice. Mol. Psychiatry.

[32] Higashida S., Nagai H., Nakayama K., Shinohara R., Taniguchi M., Nagai M., Hikida T., Yawata S., Ago Y., Kitaoka S. (2018). Repeated social defeat stress impairs attentional set shifting irrespective of social avoidance and increases female preference associated with heightened anxiety. Sci. Rep..

[33] Upadhyaya J., Pydi S.P., Singh N., Aluko R.E., Chelikani P. (2010). Bitter taste receptor T2R1 is activated by dipeptides and tripeptides. Biochem. Biophys. Res. Commun..

[34] Gaida M.M., Dapunt U., Hansch G.M. (2016). Sensing developing biofilms: the bitter receptor T2R38 on myeloid cells. Pathog. Dis..

[35] Zhou T., Huang Z., Sun X., Zhu X., Zhou L., Li M., Cheng B., Liu X., He C. (2017). Microglia Polarization with M1/M2 Phenotype Changes in rd1 Mouse Model of Retinal Degeneration. Front. Neuroanat..

[36] Dey A., Hankey Giblin P.A. (2018). Insights into Macrophage Heterogeneity and Cytokine-Induced Neuroinflammation in Major Depressive Disorder. Pharmaceutics.

[37] Nakagawa Y., Chiba K. (2014). Role of microglial m1/m2 polarization in relapse and remission of psychiatric disorders and diseases. Pharmaceutics.

[38] Zhang J.C., Wu J., Fujita Y., Yao W., Ren Q., Yang C., Li S.X., Shirayama Y., Hashimoto K. (2014). Antidepressant effects of TrkB ligands on depression-like behavior and dendritic changes in mice after inflammation. Int. J. Neuropsychopharmacol..

[39] Lawson M.A., Parrott J.M., McCusker R.H., Dantzer R., Kelley K.W., O’Connor J.C. (2013). Intracerebroventricular administration of lipopolysaccharide induces indoleamine-2,3-dioxygenase-dependent depression-like behaviors. J. Neuroinflamm..

[40] Koenigs M., Grafman J. (2009). The functional neuroanatomy of depression: distinct roles for ventromedial and dorsolateral prefrontal cortex. Behav. Brain Res..

