# Supplementary material for: Leucine–Histidine Dipeptide Attenuates Microglial Activation and Emotional Disturbances Induced by Brain Inflammation and Repeated Social Defeat Stress
Source: Nutrients. 2019 Sep 9;11(9):2161. doi: 10.3390/nu11092161 (PMC6770249; doi:10.3390/nu11092161)
Supplement: Supplementary file 1 [file nutrients-11-02161-s001.pdf]

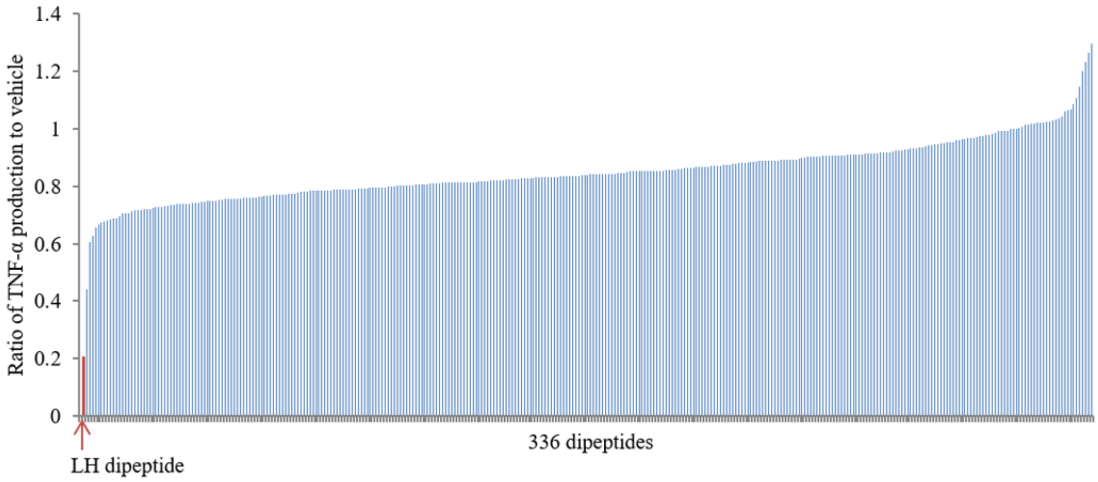

Supplementary Figure 1 Effects of dipeptides on the production of TNF-α by microglia. Effects of *in vitro* dipeptide treatment on the activation of microglia. Amount of TNF-α in the supernatants of microglial cultures pretreated with 50 μM each of 336 dipeptides and followed by treatment with 5 ng/mL LPS and 0.5 ng/mL IFN-γ. Data are expressed as the ratio of TNF-α + each dipeptide levels to vehicle treatment.
